# Anaphylactic Reaction to Cyanocobalamin: A Case Report

**DOI:** 10.7759/cureus.2582

**Published:** 2018-05-05

**Authors:** Muhammad Wajih Ullah, Afshan Amray, Aisha Qaseem, Tariq Siddiqui, Temoor Naeem

**Affiliations:** 1 Cardiology, Mayo Clinic, Rochester, USA; 2 Internal Medicine, Dow Medical College, Dow University of Health Sciences (duhs), Karachi, PAK; 3 Internal Medicine, Ziauddin Medical University, Karachi, PAK; 4 Internal Medicine, Maharashtra Institute of Medical Education & Research, Talegaon, IND; 5 Internal Medicine, Fauji Foundation Hospital, Rawalpindi, Punjab, PAK

**Keywords:** vitamin b12 deficiency, cyanocobalamin injection anaphylactic reaction

## Abstract

Vitamin B12 is essential for the development of healthy nerves and red blood cells. Vitamin B12 deficiency is becoming widespread and most commonly affects elderly, pregnant women, vegetarians, and patients with renal or intestinal diseases. Either parenteral vitamin B12 treatment or high-dose oral vitamin B12 treatment is an effective therapy regardless of etiology. Parenteral therapy using the intramuscular route is considered the most familiar treatment for vitamin B12 deficiency. Anaphylactic reaction after intramuscular injection is an uncommon and potentially serious side effect. In this study, we are documenting a case of anaphylactic reaction in a 55-year-old woman after her second dose of intramuscular injection of cyanocobalamin. The purpose of this case report is to highlight the need to understand the rare life-threatening side effect of intramuscular cyanocobalamin. Health care providers should be vigilant while administering the intramuscular injection of cyanocobalamin to vitamin B12 deficient patients.

## Introduction

Vitamin B12 is a water-soluble vitamin and is essential for the growth of healthy nerves and red blood cells. Vitamin B12 is naturally present in some foods and added to others. Although vitamin B12 is easily accessible as a dietary supplement and a prescription medication its deficiency is becoming widespread. Elderly, pregnant women, vegetarians, and patients with renal or intestinal diseases are some risk groups most likely to suffer from vitamin B12 deficiency [1]. According to American Family Physicians, intramuscular injection of 1 mg weekly of cyanocobalamin for eight weeks, followed by 1 mg monthly for life or high oral dose of 1-2 mg/dl of vitamin B12 daily for life are used to treat vitamin B12 deficiency regardless of etiology [2]. Traditionally vitamin B12 deficiency has been treated with intramuscular injection of cyanocobalamin [3]. In this case report, we are documenting a case of anaphylactic reaction in a 55-year-old woman after her second dose of cyanocobalamin intramuscularly.

## Case presentation

A 55-year-old patient presented in the outpatient department with complaints of weakness, tiredness and mild shortness of breath for four to five months. She had no associated symptoms such as fever, cough, sensory abnormalities, paresthesia, and difficulty in adjusting to surrounding temperature. She did not have any antecedent infection. Her past medical and surgical history was unremarkable. She had no known allergic reaction to food or drugs. A detailed history revealed the patient had been on a vegan diet for the past five years without taking any multivitamins or fortified food.

Her vital signs on examination were: a) Temperature: afebrile, b) Blood pressure: 135/85 mmHg, c) Respiratory rate: 15 breaths/min, d) Heart rate: 100 beats/min. General examination revealed pallor of the sclera. Her systemic examination including the neurological examination was unremarkable.

Her complete blood count revealed hemoglobin = 6.22 mg/dl (normal range: 12-15.5 mg/dl in women), reticulocyte count = 0.7% (normal range: 0.5-1.5%), red blood cells = 3.40 million/cumm (normal range: 3.70-5.60 million/cumm in women), white blood cells = 8,600/cumm (normal range: 4000-11,000/cumm), platelets = 304,100/cumm (normal range: 150,000-450,000/cumm), mean corpuscular count (MCV) = 105 fl/red cell (normal range: 80-96 fl/red cell).

Peripheral blood film revealed macrocytes with hypersegmented neutrophils. Her serum vitamin B12 levels = 142 pg/ml (normal range: 200-600 pg/ml) and plasma folate levels = 9 ng/ml (normal range: 2-20 ng/ml). Further testing revealed elevated plasma homocysteine and methylmalonic acid levels. A diagnosis of megaloblastic anemia secondary to vitamin B12 deficiency was made. Serum intrinsic factor antibodies were negative on testing.

The patient was educated about her disease and was counseled on the use of taking multivitamins and fortified food while on the vegan diet. As the patient was symptomatic, she received an intramuscular injection of 1 mg cyanocobalamin on the day of the visit and tolerated it well. She was scheduled for intramuscular injection of cyanocobalamin 1 mg weekly for seven successive weeks. However, during her second week of treatment with 1 mg cyanocobalamin intramuscularly, the patient developed generalized urticaria with pruritus, abdominal cramps, vomiting, tongue swelling and difficulty in breathing five to ten minutes.

The patient was immediately transferred to the emergency department of the hospital. On physical examination, the patient was in acute distress. Her vital signs were: a) Temperature: afebrile; b) Blood pressure: 68/52 mm Hg; c) Respiratory rate: 33 breaths/minute; d) Heart rate: 120 beats/minute. On inspection of the body, urticaria of the palms, soles and inner thighs were noticed. Chest auscultation revealed diffuse wheezes bilaterally. Abdominal examination revealed abdominal distension. She was immediately given an intramuscular injection of epinephrine with intravenous hydrocortisone. She was also given antihistamines for symptomatic treatment.

Once the patient stabilized she was scheduled the following week for a follow-up. On her visit, she was offered a high oral dose, 2 mg/dl daily, of vitamin B12. She tolerated the oral vitamin B12 well and did not report any unwanted side effects. After a few months of taking oral vitamin B12, her hemoglobin and hematocrit improved, and the patient became asymptomatic.

## Discussion

The allergic reactions due to vitamin B12 injection can be due to multiple reasons. Lagerholm et al. documented a case of hypersensitivity to benzyl alcohol added as the preservative, resulting in urticaria after injection [4]. Bedford observed hypersensitivity was due to carry out impurities formed during biosynthesis of vitamin B12, acting as antigens and causing allergic reactions [5]. According to Hovding, sensitization was due to cobalamin molecule or polypeptide bound to the cobalamin constituting a complete antigen in its own way and resulting in an allergic reaction [6].

The allergic reaction ranges from simple urticaria, and pruritus to anaphylactoid type reaction, sometimes contributing to the death of the patient. Four types of cobalamin exist - cyanocobalamin, hydroxocobalamin, methylcobalamin, and adenosylcobalamin. Both oral and parenteral formulations have been reported to induce an allergic reaction [7, 8]. The cyanocobalamin and hydroxocobalamin exist in injectable forms and are highly purified forms of cobalamin. Hypersensitivity after intramuscular injection of either cyanocobalamin or hydroxocobalamin can be due to one of the three reasons described earlier.

The hypersensitivity reaction can occur within minutes, few hours, days, months or more commonly, years after the sensitization dose of vitamin B12 [9]. In our patient, she tolerated her first dose of intramuscular cyanocobalamin. However, it sensitized the patient. When the patient was given her second dose of cyanocobalamin intramuscularly a week later, an anaphylactic reaction was induced due to type I hypersensitivity. Once the patient was switched to oral vitamin B12, she tolerated it very well and experienced no side effects. After few months of treatment with oral vitamin B12, her condition improved, and her hemoglobin normalized. The reason for tolerating the oral preparation of vitamin B12 can be explained by the absence of carryout impurities or any preservatives in oral preparations of vitamin B12.

## Conclusions

Only a few cases of anaphylactic reaction after intramuscular injection of cyanocobalamin have been reported. The purpose of this case report is to highlight the need to understand the rare life-threatening side effect of intramuscular cyanocobalamin. Health care providers should be watchful while administering the intramuscular injection of cyanocobalamin to vitamin B12 deficient individuals.
